# Industrial Fault Detection Employing Meta Ensemble Model Based on Contact Sensor Ultrasonic Signal

**DOI:** 10.3390/s24072297

**Published:** 2024-04-04

**Authors:** Amirhossein Moshrefi, Hani H. Tawfik, Mohannad Y. Elsayed, Frederic Nabki

**Affiliations:** 1 Department of Electrical Engineering, Ecole de Technologie Supérieure, ETS, Montreal, QC H3C 1K3, Canada; frederic.nabki@etsmtl.ca; 2 MEMS-Vision International Inc., Montreal, QC H4P 2R9, Canada; hani.tawfik@mems-vision.com (H.H.T.); mohannad.elsayed@mems-vision.com (M.Y.E.)

**Keywords:** fault detection, ultrasonic signal, feature extraction, meta classification, machine learning, real-time monitoring

## Abstract

Ultrasonic diagnostics is the earliest way to predict industrial faults. Usually, a contact microphone is employed for detection, but the recording will be contaminated with noise. In this paper, a dataset that contains 10 main faults of pipelines and motors is analyzed from which 30 different features in the time and frequency domains are extracted. Afterward, for dimensionality reduction, principal component analysis (PCA), linear discriminant analysis (LDA), and t-distributed stochastic neighbor embedding (t-SNE) are performed. In the subsequent phase, recursive feature elimination (RFE) is employed as a strategic method to analyze and select the most relevant features for the classifiers. Next, predictive models consisting of k-Nearest Neighbor (KNN), Logistic Regression (LR), Decision Tree (DT), Gaussian Naive Bayes (GNB), and Support Vector Machine (SVM) are employed. Then, in order to solve the classification problem, a stacking classifier based on a meta-classifier which combines multiple classification models is introduced. Furthermore, the k-fold cross-validation technique is employed to assess the effectiveness of the model in handling new data for the evaluation of experimental results in ultrasonic fault detection. With the proposed method, the accuracy is around 5% higher over five cross folds with the least amount of variation. The timing evaluation of the meta model on the 64 MHz Cortex M4 microcontroller unit (MCU) revealed an execution time of 11 ms, indicating it could be a promising solution for real-time monitoring.

## 1. Introduction

**Figure 1:**
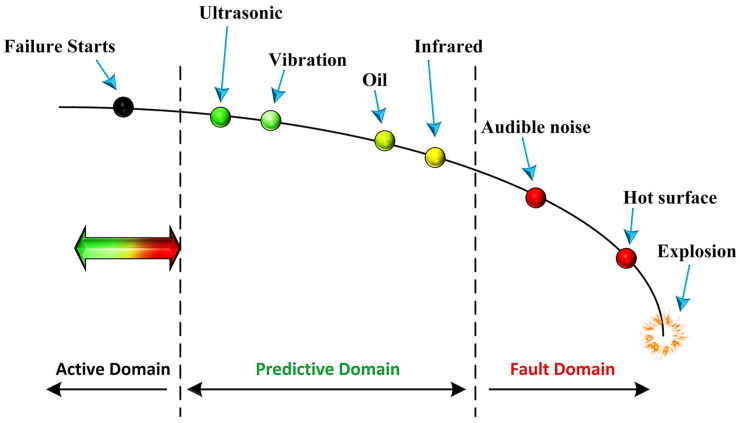
IPF curve for industrial fault prediction delay [1].

In today’s industry, rapid growth has led to more widespread automated processes, as well as an increased demand for advanced equipment and machines. Motor and pipeline spare parts have been increasingly used in machine maintenance due to the advent of industrial automation. Meanwhile, preventing the breakdown of these types of equipment would be beneficial to reduce the cost and time of maintenance. There are some methods to detect industrial faults which have different characteristics. Industrial equipment fault monitoring with technologies like vibration analysis and temperature sensing has been available for many years. However, as demonstrated by the installation potential failure (IPF) curve in Figure 1, ultrasound is alternatively an efficient technology to sense certain mechanical and electrical faults in order to predict failures much earlier than other monitoring technologies [1].

In this study, common faults of pipes and motors are investigated. In order to transport fluids from one place to another, pipes are widely used. Numerous pipe segments connected with joints make up the pipeline networks that extend for several kilometers. Various factors, including traffic and surface loads, may cause the pipes and joints in these pipelines to be overstressed, resulting in leaks and pipe bursts. On the other hand, due to pipeline defects [2] such as cracks, cavitation, corrosion, and other mechanical damage, the problem of pipeline operation safety can become very difficult to manage [3,4]. 

Moreover, bearings need to be considered for diagnosing rotating machinery faults or diagnosing shaft failures in industrial processes. Bearings are among the most important peripheral mechanisms in most developing countries and have to operate under high loads and speeds. Their failure, mostly due to incorrect lubricant selection, contamination, loss of lubricant, or over-greasing, causes malfunctioning of the machinery and shutdown, which in turn affects the quality and cost of the products [5].

Highlighting the symbiotic relationship between motors and pipelines in industrial settings is crucial for understanding their collective impact on system efficiency and safety. These components frequently operate as part of a unified system across numerous industrial applications. For instance, in the chemical processing sector, motors power pumps that facilitate the movement of fluids through pipelines. A malfunction in the motor could result in reduced pressure or flow within the pipelines, compromising the efficiency and safety of the process.

Moreover, simultaneous fault detection in both motors and pipelines fosters an all-encompassing approach to predictive maintenance. Through diligent monitoring of these components, industries can avert unplanned downtime, refine maintenance schedules, and bolster system reliability. This strategy is particularly vital in sectors such as oil and gas, where the failure of any component might induce substantial production setbacks and environmental risks. Additionally, the interconnected nature of motor and pipeline failures—where a fault in one could exacerbate issues in the other—underscores the im-portance of understanding these relationships for effective fault diagnosis and mitigation. For example, a blockage in a pipeline might overload a motor, causing it to overheat and fail, whereas motor failure could disrupt fluid flow, leading to increased pressure and strain on pipeline joints or seals. In scenarios where motors propel compressors or pumps linked to pipelines, like HVAC systems in large buildings or water treatment facilities, maintaining the faultless operation of both elements is imperative for energy conservation and cost-efficiency.

Adherence to regulatory and safety standards frequently necessitates extensive monitoring and fault detection across all crucial industrial system components. In the pharmaceutical industry, preserving the integrity of motors and pipelines is essential for ensuring product sterility and quality. Any defects in these elements could jeopardize the entire production cycle, potentially resulting in regulatory breaches and health risks. For example, in the food and beverage industry, motors drive pumps and agitators that convey ingredients, mixtures, or final products through pipelines. Failures in these systems may cause contamination, product wastage, or safety issues, highlighting the importance of unified fault detection mechanisms [6]. 

To address the aforementioned faults, many approaches have been investigated in the literature. In addition to ultrasound and vibration, other signals such as infrared and audible noise as well as oil leakage can detect faults. As discussed before, among them, ultrasound signals have the ability to distinguish industrial faults more efficiently [7]. However, in order to analyze ultrasonic signals, conventional methods like FFT [8], filtering, and windowing [9,10], due to the overlapping of noise and signal, are not effective. Alternatively, intelligent methods like machine learning (ML) can detect subtle changes in the received signals from the ultrasonic sensor [1,11].

Based on the literature, many authors studied fault detection using artificial intelligence (AI) algorithms by focusing on various derived signals. Quy et al. [3] introduced a technique using a KNN classifier embedded in a microcontroller unit to classify the leak faults in real-time. They extracted the hybrid features from acoustic emission signals through a gas pipeline. Ding et al. [12] introduced an algorithm for detecting faults in spacecraft, specifically focusing on four types of leaks with varying leakage conditions. Their approach utilizes acoustic signals and combines SVM with empirical mode decomposition (EMD) as the underlying technique. According to the assessment of a pipeline by Coelho et al. [13], a system based on a wireless sensor network designed to monitor distribution systems using a random forest (RF) algorithm was used to precisely locate fluid leaks. They concluded that, by employing more features, accuracy could be increased. Rai et al. [14] highlighted the insufficiency of historical data on pipeline failures for the existing supervised AI methods. To address this challenge, they introduced an alternative approach focused on a health index perspective. Their proposed method incorporates multiscale analysis, the Kolmogorov–Smirnov (KS) test, and a Gaussian mixture model (GMM) to accurately determine the leakage situation in pipelines.

Heng et al. [15] proposed a method that employs Principal Component Analysis (PCA) to integrate data from multiple sensors for the accurate prediction of rolling bearing lifespan. This approach significantly enhances reliability by amalgamating various features from vibration signals, though it relies heavily on extensive lifecycle data for effective modeling. A notable limitation of their work is its dependence on complete lifecycle test data from rolling bearings to achieve precise modeling, necessitating comprehensive testing to identify optimal sample sizes. This requirement potentially limits the method’s application in scenarios where extensive historical data is unavailable, likely increasing the time and cost associated with the predictive maintenance process. Additionally, this method primarily focuses on time-domain features, which may restrict the depth of data feature analysis and risk missing valuable insights from other domains, such as frequency. 

Jose et al. [16] introduced a condition monitoring methodology for induction motors, analyzing motor stator currents and vibrations to estimate different features across multiple domains. Utilizing genetic and PCA algorithms for feature selection, followed by dimensionality reduction via the Linear Discriminant Analysis (LDA) algorithm, this approach ultimately evaluates the refined features using a neural network classifier to achieve global and individual classification ratios. However, the complexity of this method and its intensive computational resource requirements are significant drawbacks. The process involves multiple advanced stages, including high-dimensional feature estimation, optimization with Genetic Algorithms and PCA, and dimensionality reduction through LDA, before proceeding to fault diagnosis. This complexity not only necessitates considerable processing power but also complicates real-time application, potentially limiting its practicality in scenarios where rapid and efficient diagnostics are essential.

Research on rotating machinery fault diagnosis using ensemble kernel extreme learning machines based on stacked denoising autoencoders (SDAE) was explored [17]. This study focused on gear, rotor, and real engine rolling bearing datasets, extracting vibration characteristics from both time and frequency domains. The PCA algorithm was used to merge two sets of SDAE features from multiple domains into essential low-dimensional features, which were then classified for fault patterns using the ensemble kernel extreme learning machine. Despite its innovative approach, this method encountered challenges such as lengthy training times, complex implementation, high computational costs, and the need for large amounts of labeled data for training the models.

Our contribution builds upon these insights, utilizing ultrasonic data for early detection characterized by its minimal computational demands. This enables efficient deployment on MCUs, offering quick responsiveness with reduced memory usage, thereby addressing some of the limitations highlighted in the aforementioned studies.

Maliuk et al. [18] conducted research on detecting bearing faults through signal processing and proposed another approach based on the Gaussian mixture model (GMM). Their method leverages a fault-frequency-oriented GMM window series for reliable feature extraction. The classification step is performed using the weighted KNN algorithm. As discussed before, besides pipeline faults in industrial equipment, rotating machinery faults commonly occur in many different areas and manufacturing sectors. These include aerospace, power generation, oil refining, machining, automotive, railway transportation, pumping systems, etc. [6,19,20]. To prevent operational malfunctions that could potentially result in catastrophic failures, various condition monitoring techniques have been developed for the purpose of fault detection and diagnosis in bearings. Abdelrhman et al. [21] introduced a diagnosis and detection model for bearing faults in rotating machinery. Their approach involves the utilization of multivariate analysis of variance to extract parameters from acquired data sets. Time-domain features are employed, and a binary logistic regression (BLR) modeling technique is utilized for the fault diagnosis and detection. Similarly, Jiang et al. [22] proposed a method for weak rotating machinery fault diagnosis. They introduced a multiscale permutation entropy feature extraction approach, which involves calculating time series with equal overlapping segments. The extracted features are then used as input for classification using SVMs. To optimize the SVM parameters, the authors introduced a chaos firefly optimization algorithm as a solution to the parameter optimization problem. Another study by Souza et al. [23] proposed an SVM-based classifier by employing statistical data features for bearing fault recognition. They investigated the presence of different types of bearing defects and combinations of statistical attributes on SVM accuracy. In another work by Jung et al. [24], using adaptive wavelet denoising and statistical–spectral acoustic features, the authors performed a binary classification task to monitor the health conditions of a ball bearing with a microphone under noisy conditions. The authors used an adaptive wavelet method based on the kurtosis entropy index and multiple acoustic features were extracted based on expert knowledge. 

However, few works have investigated the use of an ensemble model in fault detection though ultrasonic monitoring. In [25], we proposed a meta model based on the majority voting of four classifiers for industrial faults. Accordingly, this study is an extension of previous work and focuses on fault diagnosis using ultrasound signals by a meta ensemble model. The research encompasses the classification of ten distinct types of faults in both pipeline and rotating machinery conditions. Statistical features from both the time and frequency domains are extracted by the PCA, LDA, and t-SNE approaches. Finally, the features are refined using RFE. Then, five widely used classification models including GNB, LR, KNN, SVM, and DT are implemented. Afterward, using an ensemble learning approach, faults are classified into ten categories. Then, the reliability of the model based on four evaluation metrics—k-fold validation, confusion matrix, ROC, and learning curve—is investigated and, ultimately, its potential for real-time application is assessed through deployment on an MCU. 

Our study makes significant contributions by meticulously preparing a dataset that encapsulates 10 distinct fault types, integrating both motor and pipeline anomalies. In the initial preprocessing phase, we employ advanced techniques to effectively eliminate noise, enhancing the data’s clarity. We then leverage feature reduction methods, including RFE, to meticulously select the most impactful features for subsequent analysis. During the classification phase, we introduce a novel approach by combining conventional classifiers into an innovative ensemble model. This model is rigorously evaluated through k-fold cross-validation, demonstrating its ability to accurately categorize features with 93% accuracy. The culmination of our efforts is the deployment of this framework on an MCU, where we successfully assess its capability for real-time monitoring. This comprehensive approach not only showcases the framework’s high accuracy but also its practical applicability in real-world industrial settings for fault detection.

The rest of this paper is organized as follows: preprocessing, feature extraction, and methods are explained in Section 2. Then, Section 3 presents the experimental results and is followed by a conclusion.

## 2. Proposed Methodology

**Figure 2:**
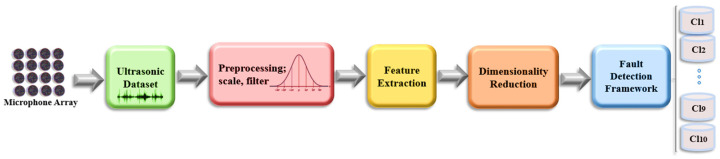
Overview of the workflow methodology.

The proposed methodology’s overview is illustrated in Figure 2. The entire procedure consists of five steps, each of which is explained in a separate subsection. The initial step involves acquiring ultrasonic data from a microphone array module, which is then recorded as raw data for further classification analysis. The collected data from the structure is processed to refine and prepare it for the feature extraction stage. Feature extraction is performed to transform the data into a set of numerical features known as a feature vector, which provides a concise and informative representation of the data. This study extracts a comprehensive suite of features from ultrasonic data, spanning time, power spectral density, and frequency domains. These features include minimum, maximum, mean, zero crossing times, slope change, impulse factor, shape factor, margin factor, area under curve, standard deviation, skewness, and kurtosis, offering a detailed analysis of the data’s characteristics. The presence of large input vectors often poses a challenge in the predictive modeling process of machine learning models. This challenge is commonly known as the problem of high dimensionality in raw data. To address this, a dimensionality reduction technique is employed as a solution. Finally, a machine learning classification algorithm is applied to the reduced-dimensional data for the classification task.

### 2.1. Preprocessing

To mitigate noise in ultrasonic signals, one technique involves utilizing a Butterworth filter in the time domain, aimed at eradicating undesired spikes, trends, and outliers. Butterworth filters are favored in control systems for their lack of peaking, ensuring a consistent signal without unduly amplifying any frequency. These filters are crafted to sustain a flat frequency response across the passband, avoiding alterations in signal amplitude within this range [26]. This characteristic is crucial for maintaining the integrity of the ultrasonic signal’s information content. The nth order Butterworth filter is mathematically described by its frequency response equation, encapsulating the filter’s design to achieve the desired signal such that the following holds:

(1) $$H_{\left(j\omega \right)}=\frac{1}{\sqrt{1+{\varepsilon^{2}(\frac{\omega }{\omega_{p}})}^{2n}}}$$

where H is the passband gain, ω is the angular frequency, ε is the maximum pass band gain, and n represents the filter order. 

The data values are normalized to be comparable for the different signal sets. Among many different scalers available for feature scaling, the standard scaler in [27] is normalized as follows:

(2) $$Z=\frac{X-\mu }{\sigma }$$

where the normalized vector, denoted as Z, is derived from the signal vector X. The normalization process involves calculating the mean (μ) and standard deviation (σ) of the input acoustic sequence X. These statistical measures are used to standardize the values in X, resulting in the normalized vector Z.

### 2.2. Feature Extraction

Firstly, to provide the characteristics of each data, some features should be considered. In this study, 30 features in time, Fourier, and power spectrum domains are extracted which are composed of maximum, average, standard deviation, skewness, kurtosis, average of peaks (peaks_mean), time of peak (peak_index), zero crossing, crest factor, and shape factor. To distinguish between the classes with the most discernment, the independent and more important features should be selected. The selected complex features are defined below:

(3) $$Skewness=\frac{\frac{1}{N}\sum_{n=1}^{N}(x(n)-\bar{x}{)}^{3}}{(\sqrt{\frac{1}{N}\sum_{n=1}^{N}(x(n)-\bar{x}{)}^{2}}{)}^{3}}$$

(4) $$Kurtosis=\frac{\frac{1}{N}\sum_{n=1}^{N}(x(n)-\bar{x}{)}^{4}}{(\sqrt{\frac{1}{N}\sum_{n=1}^{N}(x(n)-\bar{x}{)}^{2}}{)}^{4}}$$

(5) $$Crest\_factor=\frac{\max|x(n)|}{\sqrt{\frac{1}{N}\sum_{n=1}^{N}x(n{)}^{2}}}$$

(6) $$Shape\_factor=\frac{\sqrt{\frac{1}{N}\sum_{n=1}^{N}x(n{)}^{2}}}{\frac{1}{N}\sum_{n=1}^{N}\left|x(n)\right|}$$

where N is the number of samples in the input vector *x*(*n*).

### 2.3. Dimensionality Reduction

In order to enhance the accuracy and efficiency of the model, it is essential to prune every dataset. Particularly when dealing with ultrasonic signals, the dimensionality of the data should be reduced. Dimensionality reduction involves transforming high-dimensional data into a more concise and meaningful representation with reduced dimensions. One of the prominent techniques for linear dimensionality reduction is PCA which identifies orthogonal directions that capture the maximum variance, making it a valuable second-order statistical method [28]. PCA is a highly effective approach for eliminating redundant dimensions while retaining the most informative ones. This algorithm operates by computing the eigenvalues of the covariance matrix, aiming to minimize computation costs while preserving accuracy. By extracting independent information from the acoustic data, PCA successfully reduces dimensionality while maintaining the essential aspects of the dataset. In addition to PCA, another algorithm commonly used for assessing feature distinctiveness and visualizing data separability is t-SNE, which is capable of preserving the relationships among data points in a lower-dimensional space, making it particularly useful for visualizing complex high-dimensional data. One key distinction between the PCA and t-SNE techniques is that while PCA focuses on preserving large pairwise distances to maximize variance, t-SNE primarily preserves local similarities among data points. This difference in approach makes t-SNE well-suited for visualizing intricate patterns in the data [29,30]. For enhanced comparison and visualization, LDA, a supervised technique, is utilized. LDA seeks to generate new components that differentiate categories effectively, focusing on a class feature with the goal of identifying the projection that optimally distinguishes between faults in the ultrasonic data [31]. By reducing the dimensionality of the data, LDA strives to improve class separability while retaining as much information as possible. Although it offers a solution to the challenge of small sample sizes, LDA faces issues such as singularity, dependence on the assumption of Gaussian distributions, and the difficulty in determining the best dimensionality reduction.

### 2.4. Recursive Feature Elimination (RFE)

**Figure 3:**
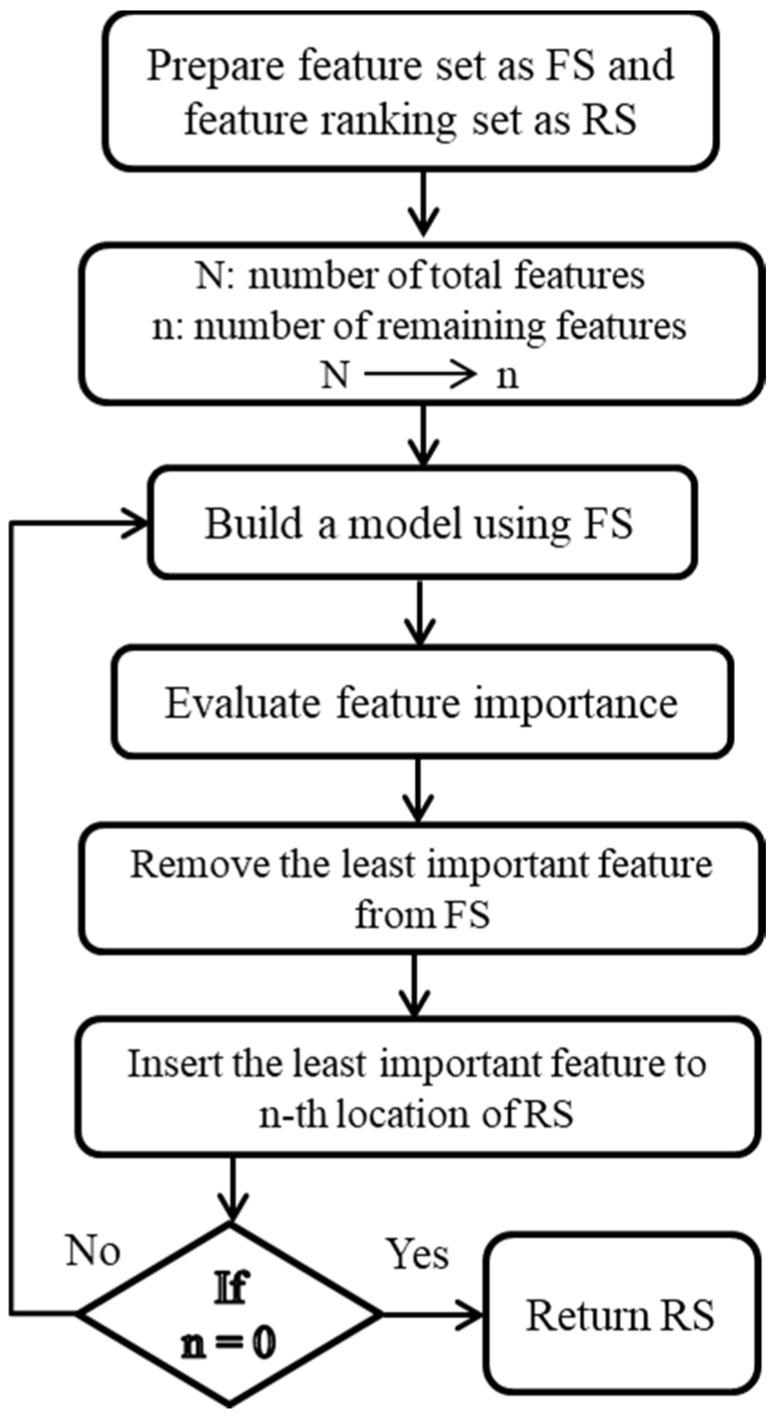
Recursive feature elimination flow diagram.

RFE selects features recursively, considering increasingly smaller sets of features at each step. By comparing different feature sets, the algorithm aims to identify the set that achieves the highest accuracy [32,33]. In every iteration of the RFE algorithm, the importance of each feature is measured, and the least relevant feature is subsequently removed. In other words, RFE aims to evaluate the significance of features within a dataset, given an external estimator or classifier that assigns importance weights to these features. This method systematically selects ultrasonic features from the feature set (FS) by progressively focusing on increasingly smaller subsets of ultrasonic characteristics through iterative processes. Features that are deemed less important and thus eliminated in each iteration are then placed into a ranking set (RS). With each subsequent iteration, the FS is methodically reduced to further assess the importance of the remaining features. This procedure is repeated multiple times to exhaustively analyze all possible combinations of features within the FS. Ultimately, the features are systematically organized and ranked within the RS based on their importance, as determined through the RFE algorithm. The RFE procedure is depicted in Figure 3.

### 2.5. Classification Using k-Fold Cross Validation

In this study, for an industrial fault detection system, we propose an effective monitoring method using ultrasonic signals based on well-known classifiers. The classification algorithm first obtains raw signals, and it then preprocesses and scales the time series ultrasonic signals. Consequently, a variety of time- and frequency-domain features are extracted from the data, which serve as input to the classifier. Then, five classifiers including KNN, LR, DT, GNB, and SVM are employed to distinguish the fault classes. The selection of these classifiers is based on their reduced memory requirements with lower output variations in comparison to deep learning classifiers [34], making them well-suited for ensemble implementation. Afterwards, a meta classifier based on k-fold cross validation (CV) is designed to detect and classify the irregularities in the ultrasonic signals. The mentioned classifiers are described as follows:

SVM is a clustering algorithm specifically designed for small sample sets, demonstrating exceptional learning capabilities even when the data is limited. It also exhibits good generalization properties. The SVM algorithm, utilizing a kernel function, aims to identify the optimal hyperplane or decision boundary (such as a point, line or plane) that effectively separates different classes of data [35].

GNB algorithm is an extension of the Naive Bayes algorithm. Naive Bayes is a generative model that assumes the independence of features. In the case of Gaussian Naive Bayes, the algorithm assumes that the covariance matrices are diagonal, implying independence between the features. Different functions are employed to estimate the data distribution and calculate the mean and standard deviation for the training [36].

LR is a machine learning classification algorithm widely used in statistical analysis. It is employed to describe data and model the relationship between a single dependent variable and two or more independent variables, which can be of ordinal, nominal, interval, or ratio level. The purpose is to determine the outcome or predict the probability of a specific event or class [37].

DT is a supervised machine learning algorithm used for both regression and classification tasks. It is constructed using a directed graph technique. The decision tree follows a tree-like structure, where inner nodes represent the variables or features of a dataset, branches represent the decision rules, and each leaf node represents the output result [38].

**Figure 4:**
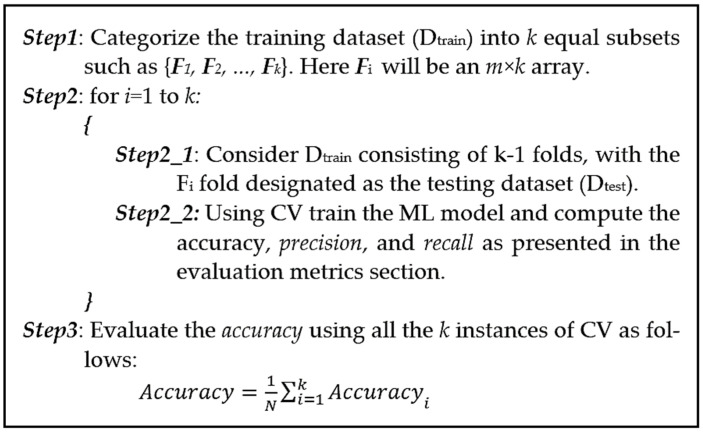
Pseudo-code for the k-fold CV algorithm.

KNN is a supervised classification approach that avoids making any assumptions about the primary data. It is a non-parametric algorithm that utilizes the similarity between new data and existing data to assign the new data to the most similar group among the available groups [39].

k-fold CV is employed to evaluate the generalization of industrial fault detection analysis results on an independent dataset. By employing k-fold CV, we can assess the predictive performance of our machine learning model on unseen ultrasonic data and ensure the model’s robustness [40]. Using this approach, our underlying dataset is divided into k non-overlapping folds. As shown in Figure 4, in the test phase, the first ultrasonic signal fold is used to evaluate the model performance, while the remaining k−1 folds are used for training. Next, the second input signal fold is used for the test step and the rest of our dataset is used for the training. The procedure is repeated k times until the last ultra-sonic fold, and at last, the average value of these k experiments is used for the evaluation [39]. In this work, stratified k-fold CV is employed, which makes the results less susceptible to data bias and imbalance. Steps for k-fold CV are summarized as follows:

### 2.6. Meta Classifier

**Figure 5:**
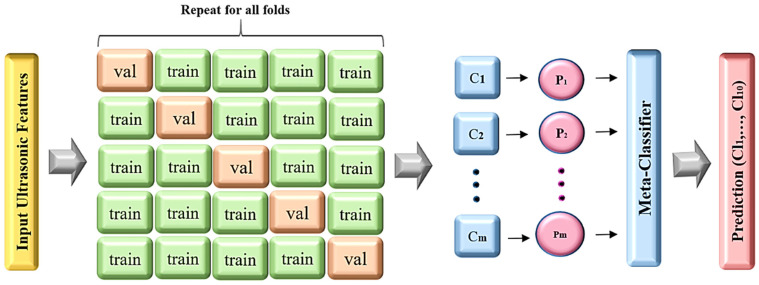
Utilization of a fault detection framework employing the k-fold CV and meta-classifier.

In the realm of ensemble learning methodologies, stacking involves the integration of multiple learning models through a meta-classifier. As illustrated in Figure 5, the initial step entails training individual classification models (C_i_) using the entire ultrasonic training set. Subsequently, the meta-classifier is trained using the labels from these individual machine learning models within the ensemble [41]. The forecasted outputs of the classifiers (P_i_) serve as meta-features, which are then combined with weights (w_i_). These weighted meta-features are utilized as inputs for the meta-classifier, ultimately determining the final output ($\hat{y}$), which is chosen based on the predicted class output from other classifiers. This process is defined as follows:

(7) $$\hat{y}=\arg\;\underset{i}{max}\sum_{j=1}^{m}w_{j}P_{ij}$$

Ensemble methods, which aggregate predictions from various base models, often en-hance generalization and robustness, outperforming single-model approaches due to the diversity in the base models. In contrast, deep learning models are susceptible to overfit-ting, particularly with limited datasets or excessive model capacity. Stacking meta-classifiers addresses this issue by integrating outputs from several models, each trained on different data subsets or utilizing distinct algorithms, thereby reducing the risk of overfit-ting.

Stacking meta-classifiers is especially advantageous for small datasets, where they tend to outperform deep learning models that typically require vast amounts of labeled data for effective training. Moreover, deep learning models demand substantial computational power and specialized hardware like GPUs or TPUs, making them less accessible. In contrast, stacking meta-classifiers are less demanding on resources and can be trained more efficiently on conventional hardware.

Therefore, stacking meta-classifiers is often a superior choice in situations demanding high interpretability, limited data availability, constrained computational resources, simplicity in model complexity, robustness against overfitting, reliability of results, and when domain expertise is a key factor.

### 2.7. Implementation

The “micromlgen” library, as depicted in this study, represents a critical tool for the deployment of machine learning models on microcontrollers, facilitating the conversion of such models into C code suitable for hardware environments constrained by memory, processing power, and energy consumption. Despite its utility, the practical implementation of “micromlgen” for classification tasks reveals several limitations, particularly due to the restricted computational resources of microcontrollers. The “micromlgen” library exhibits optimal performance with simpler or specifically tailored lightweight models, given its support for a finite range of ML algorithms. This includes well-known algorithms such as decision trees, support vector machines, and certain neural network types, albeit excluding support for more complex or recent models like extensive deep learning architectures.

In the realm of TinyML, which optimizes AI for low-power, compact microcontrollers, the nRF52840 microcontroller stands out for its capability to deploy AI models efficiently, leveraging TinyML libraries. This synergy is particularly conducive for embedding on-device decision-making capabilities, thus diminishing the reliance on perpetual cloud connectivity. Utilizing “micromlgen” enables the conversion of ML models into C code, rendering it compatible with the development environment of the nRF52840. This process allows for the direct integration of AI into devices, facilitating real-time data processing and decision-making. The application of TinyML libraries further enhances model execution efficiency on the nRF52840, capitalizing on the microcontroller’s functionalities while managing its limitations.

**Figure 6:**
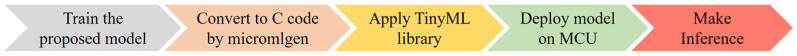
Work flow of the model implementation.

The integration of “micromlgen” for model conversion and TinyML for deployment on the nRF52840 MCU embodies a strategic methodology for embedding ML into hardware systems. This combination exploits the advantages of the aforementioned tools and the MCU’s capabilities, paving the way for the development of intelligent embedded systems proficient in edge data processing. The implementation flowchart, illustrated in Figure 6, delineates this comprehensive process, underscoring the potential for developing autonomous, cloud-independent devices that prioritize privacy, speed, and reliability.

### 2.8. Evaluation Metrics

In assessing the efficacy of the fault detection framework put forth, conventional quality metrics including precision, recall, F-measure, and accuracy are employed. These metrics furnish quantitative evaluations of the framework’s ability to identify faults, drawing from parameters such as true positive (TP), false negative (FN), true negative (TN), and false positive (FP) [42]. They are defined as follows:

(8) $$Precision=\frac{TP}{TP+FP}$$

(9) $$Recall=\frac{TP}{TP+FN}$$

(10) $$F1=\frac{2\times Precision\times Recall}{Precision+Recall}$$

(11) $$Accuracy=\frac{TP+TN}{TP+FN+TN+FP}$$

TP signifies the count of accurately classified samples deemed relevant, FN indicates the count of relevant samples inaccurately classified, TN denotes the count of accurately classified samples considered irrelevant, and FP represents the count of samples inaccurately classified as relevant. Precision quantifies the ratio of accurately classified relevant samples to all samples classified as relevant. Recall gauges the ratio of relevant samples accurately classified to all relevant samples within the dataset. The F1 score calculates the harmonic mean of precision and recall, furnishing a balanced assessment of classification performance that considers both precision and recall. Accuracy determines the proportion of correctly classified samples across all classes, presenting an overall gauge of the accuracy of classification [43]. In addition, receiver operating characteristic (ROC) plots offer a comprehensive view of a classifier’s performance across various levels of specificity. In this research, ROC plots are utilized to further investigate and evaluate the performance of the classifiers. The ROC plots provide insights into the trade-off between the true positive rate and the false positive rate, allowing for a more detailed analysis of the classifiers’ performance.

## 3. Results and Discussion

Ultrasonic fault detection for pipes and motors is a technique to identify defects or irregularities. The process involves using a contact ultrasonic microphone sensor, also known as an ultrasonic transducer. The contact ultrasonic microphone sensor is placed in direct contact with the surface of the pipe or motor to receive the data. The profile can be due to friction, impacts, leaks, or cracks that occur naturally during operation or as a result of damage. 

Healthy and uniform material emits the wave in minimal disruption. Some advantages of contact measuring are that it requires no interruption of normal equipment operation, allowing for continuous monitoring. It can identify very small or early-stage faults that might not impact visible performance or be detectable through other non-destructive testing methods. 

Also, it eliminates the cost of an external ultrasonic transmitter and the associated operational complexities. This contact sensing is particularly suited to environments where continuous monitoring is desired without the need for frequent manual inspections or where the operational noise of the equipment itself can be leveraged to identify potential issues. 

The dataset extracted from UE Systems Co. by a piezoelectric array called an “Ultraprobe” consist of 10 classes, including 4 for bearings (i.e., under-lubricated, over-lubricated, slow speed, and healthy bearing) and 6 for pipelines (i.e., steam, cavitation, motor-boating, reciprocating, thermostatic, and healthy pipe), with 20,000 samples [44], 70% for training and 30% for testing, along with a sliding window with length of 1000 to select and augment the data.

**Figure 7:**
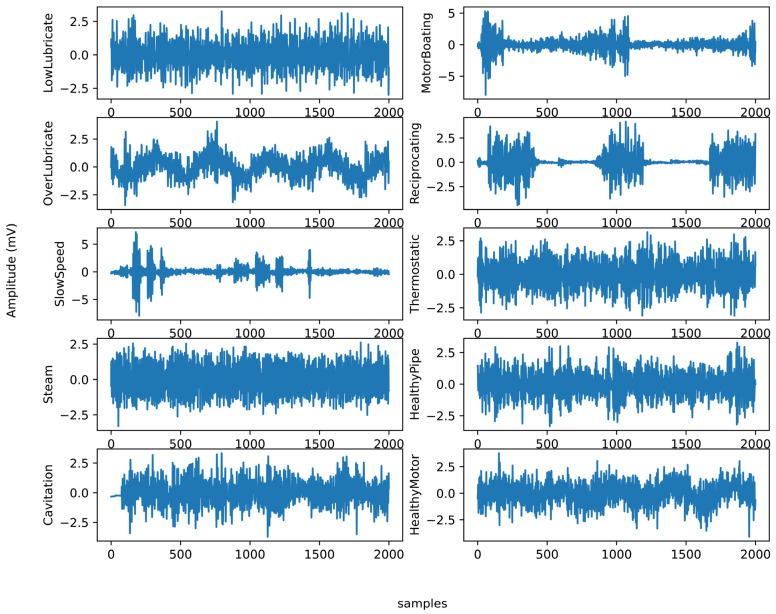
Ultrasonic signals of the ten patterns: under-lubricated, over-lubricated, slow speed, steam, cavitation, motor boating, reciprocating, thermostatic, healthy pipe, and healthy motor signals.

The motor rolling bearing and pipeline faults mainly consist of the main body which contains the ultrasonic signals of ten patterns: under-lubricated, over-lubricated, slow speed, steam, cavitation, motor boating, reciprocating, thermostatic, healthy pipe, and healthy motor signals. The data samples are shown in Figure 7.

**Figure 8:**
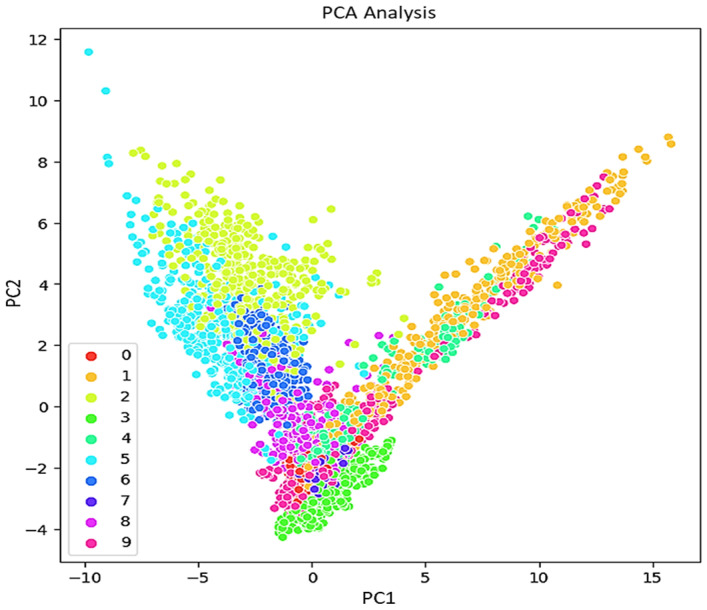
PCA representation of the ultrasonic dataset.

**Figure 9:**
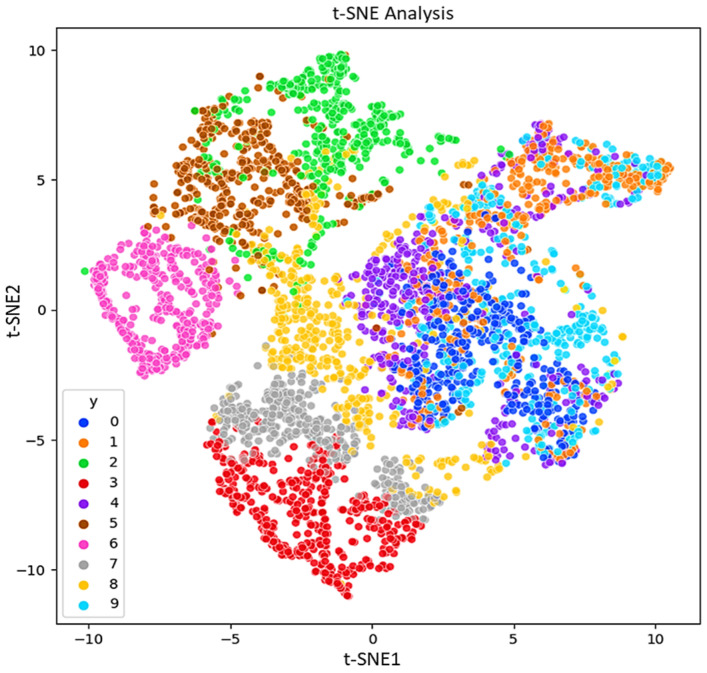
t-SNE representation of the ultrasonic dataset.

**Figure 10:**
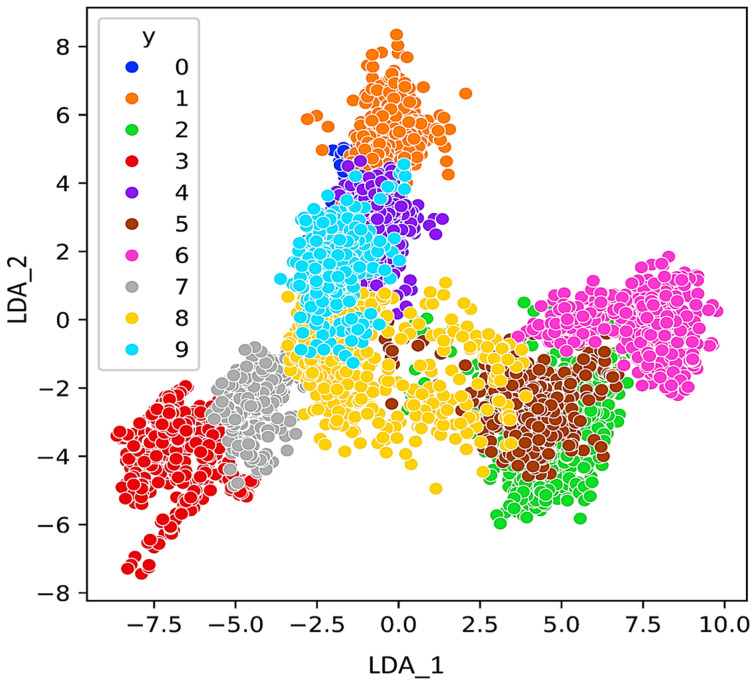
LDA representation of the ultrasonic dataset.

As mentioned in the introduction, regarding the IPF curve, the rapid identification of bearing and pipe faults is a primary objective in the operation of industrial mechanisms. This study focuses on investigating the impact of features on the statistical parameters of the acquired ultrasonic datasets. The aim is to understand how different features affect the statistical properties of the collected ultrasonic data, which are crucial for the quick identification of faults in bearings and pipes. For visualization the features, other methods like PCA, LDA, and t-SNE can be considered. PCA preserves the variance in the data and is applied to the data to reduce its dimensionality and readily visualize the faults. In addition to PCA for dimensionality reduction, t-SNE also is calculated. Figure 8, Figure 9 and Figure 10 respectively, present the PCA, t-SNE, and LDA visualization of ultrasonic data based on two components. As mentioned in the previous section, five classifiers for our model are employed. For GNB (smoothing= 0.008), DT (orientation: ”Gini”, min split sample: 3), LR (sklearn = 0.98), kNN (k = 7), and SVM (cost = 0.520 and Gaussian kernel), m = 5 are considered.

Feature selection is a critical step in the classification process, aimed at identifying and eliminating irrelevant, less useful, and redundant features, while identifying the most relevant and informative inputs for a classification model. By reducing the number of in-put features, feature selection assists in the development of a predictive model. It plays a crucial role in enhancing the efficiency and accuracy of the classification task by focusing on the most significant features that contribute to the classification performance while eliminating noise and irrelevant information. Furthermore, feature selection is desirable to both decrease the computational cost of classification models and, in some cases, to improve the performance of the fault detection model. It enables onboard ML for MCU devices by decreasing the required memory size. However, deploying ML models on MCUs presents challenges, notably in terms of processing power consumption and limitations in flash memory, which can restrict the implementation of all available features.

**Figure 11:**
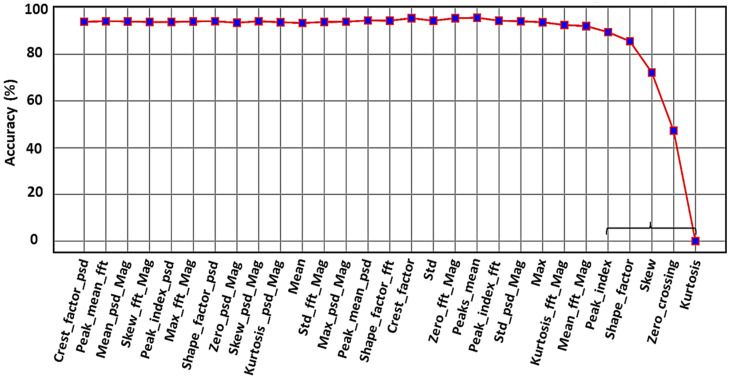
Feature selection using the RFE algorithm.

Figure 11 shows the RFE method performance for our ultrasound dataset. Mostly for implementation, the RFE results can be used to deploy our desired ML models on the MCU, and the final model is used for real-time industrial monitoring. 

In this graph, it is clear that when five features, bracketed to the right of Figure 11, are eliminated (i.e., kurtosis, zero crossing, skewness, shape factor, and peak index), the accuracy drops dramatically, indicating that these features play the more important role to distinguish the classes. 

**Figure 12:**
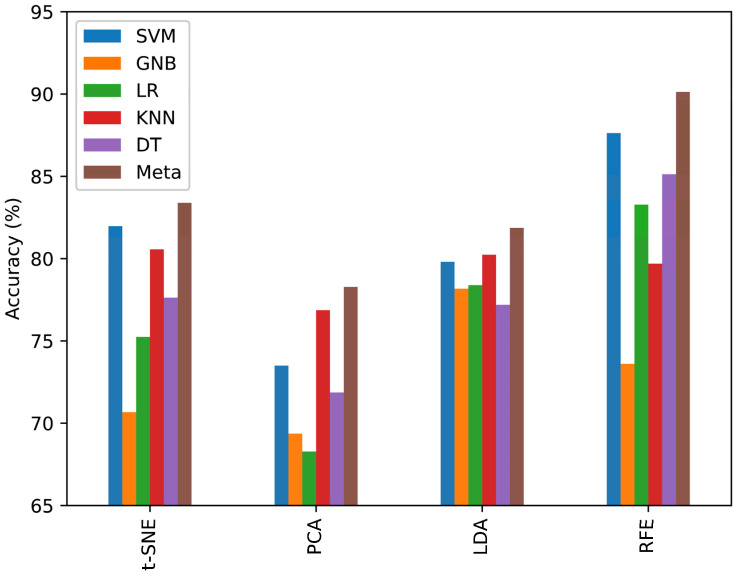
Applying multi-class classification using dimensionality reduction t-SNE, PCA, LDA, and RFE feature selection.

To validate the efficacy of the methods employed, accuracy analyses were conducted across four techniques for six classifiers. RFE distinguished itself by delivering superior accuracy in pattern classification, a claim substantiated by the results depicted in Figure 12. The comparative analysis reveals that RFE achieves commendable diagnostic outcomes relative to alternative data-reduction strategies. The metric result of the six classifiers based on RFE is shown in Table 1.

**Figure 13:**
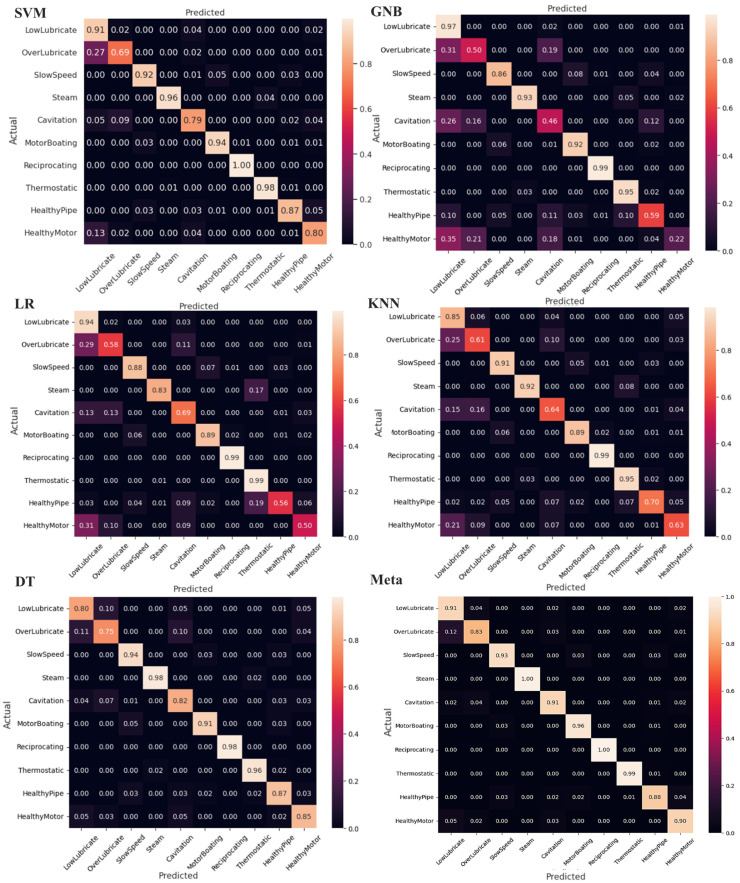
The confusion matrix based on the achieved results by the SVM, GNB, LR, KNN, DT, and meta-classifier models.

**Figure 14:**
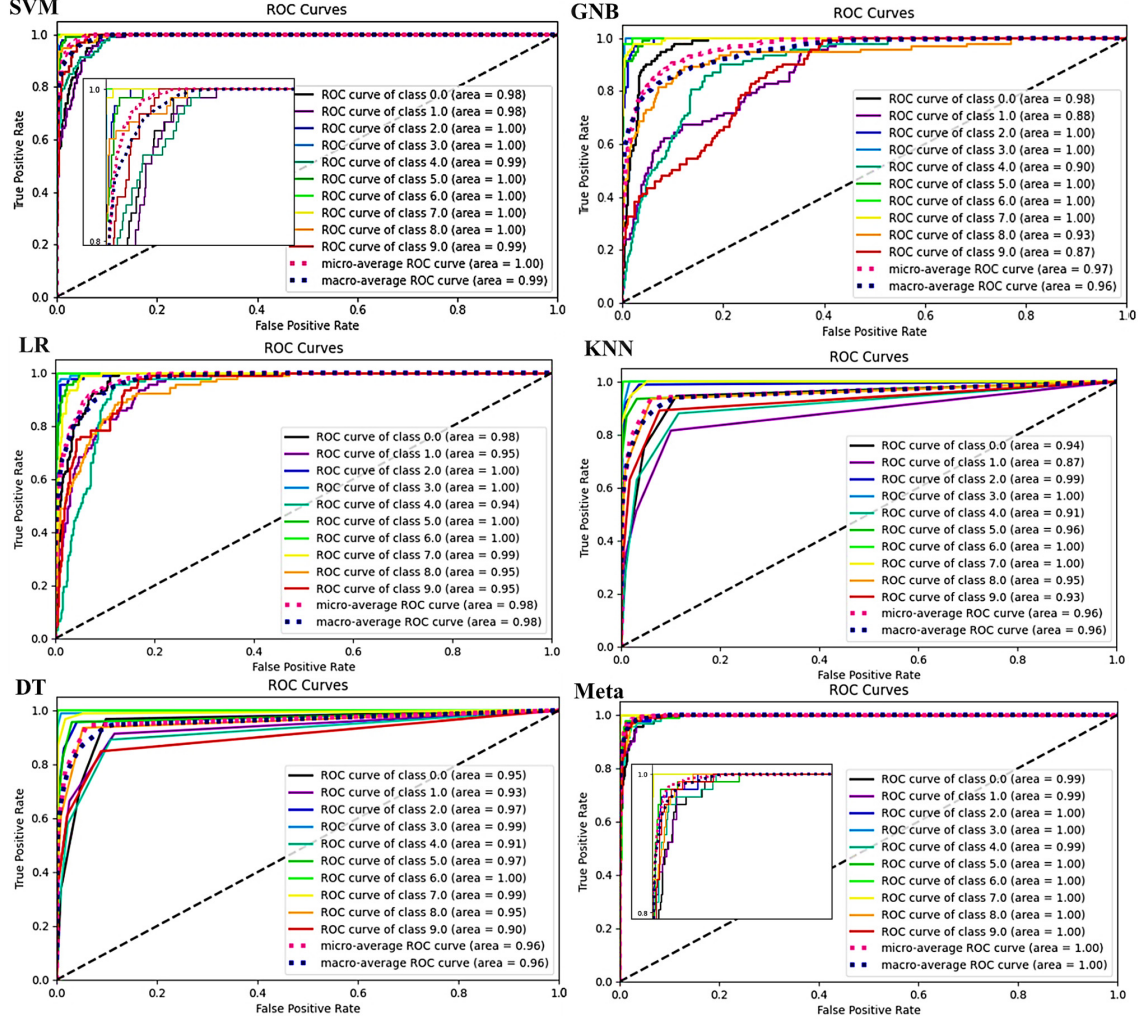
ROC curves for six classifiers.

The results in Table 1 show the superiority of the meta model for all the scores across the ten classes. The classification results of the selected methods, along with the utilized meta-classifier technique, are summarized using a confusion matrix, as shown in Figure 13. The confusion matrix provides insights into the ability of the methods to accurately classify different ultrasonic fault states. Higher values along the diagonal indicate a better model, as it correctly classifies the majority of the samples. Since the dataset used in this research includes both pipe and bearing faults, it allows us to evaluate the performance of the proposed method in handling mixed faults involving both pipes and bearings. Figure 14 illustrates the ROC curves of the selected models, utilizing k-fold CV.

The ROC curve serves as a measure of separability, indicating the capability of the selected models to distinguish between different fault classes. It provides a visual representation of the trade-off between the true positive rate (TPR) and false positive rate (FPR) for a predictive model across various probability thresholds. Basically, for every threshold, TPR and FPR are calculated and plotted on one chart. As can be seen, the lower FPR and the higher TPR for the given RFE thresholds demonstrate the better results. A steeper and more vertical ROC curve is desirable, as it indicates a model with better discriminative ability. The ROC curve analysis is instrumental in evaluating the performance and effectiveness of the selected models for fault classification tasks. In Figure 14, it can be observed that the meta model, which refers to a composite model combining predictions from multiple models, exhibits an area under the ROC curve (AUC) of 0.997 for the ultrasonic test dataset. This AUC is significantly greater than those of the other classifiers, underscoring the meta model’s superior discriminative capability and predictive performance for fault classification.

Therefore, it can be concluded that the selected meta model is reliable and demonstrates strong usability in the context of ultrasonic fault detection. Its high area under the ROC curve suggests that it can effectively distinguish between different fault classes and make accurate predictions.

**Figure 15:**
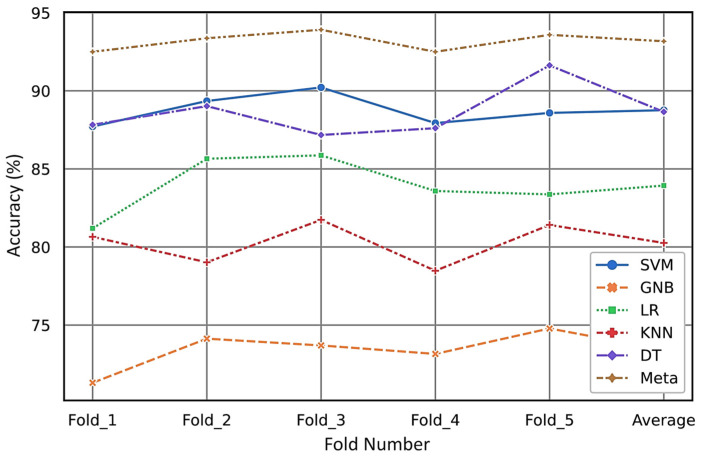
Accuracy of the six classifier models using the k-fold CV strategy.

Furthermore, in this research on ultrasonic fault evaluation, stratified k-fold CV is utilized as a method for rotation estimation to assess the generalizability of the statistical analysis to an independent ultrasonic dataset. Our simulations provide a quantitative analysis of the classification performance across five folds and the selected ML models, as presented in Table 2. The average test accuracy obtained through rotation estimation for the meta-classifier is 93%. This average accuracy of the meta model surpasses that of the other models under consideration. To better provide analysis of our simulation results over the selected models through the k-fold CV method, the accuracy of each model is visualized in Figure 15.

**Figure 16:**
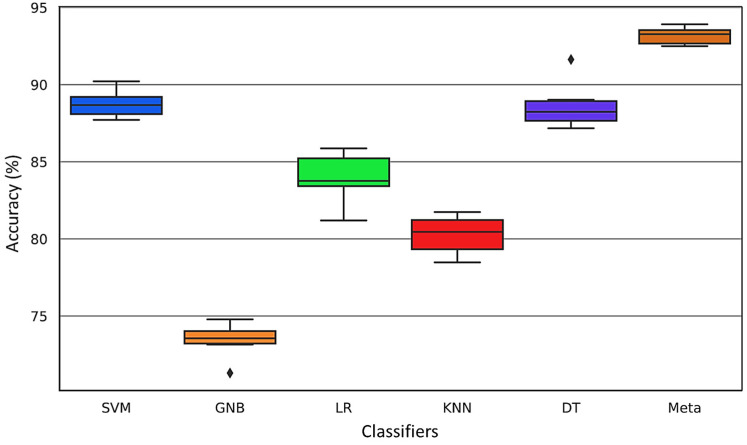
Boxplot for the six classifier models.

For more comparison, the variations in the results during the CV step for different folds are presented through a boxplot in Figure 16.

The distribution of the data allows for effective comparisons of accuracy across different folds within the ultrasonic dataset. The boxplot offers a graphical depiction of the five-number summary of the dataset, encompassing the minimum value, first quartile (25th percentile), median (50th percentile), third quartile (75th percentile), and maximum value. The ‘box’ visualizes the interquartile range (IQR), the span between the first and third quartiles, while the ‘whiskers’ extend to the furthest data points not considered outliers, which are plotted individually. This boxplot succinctly captures the distribution, central tendency, variability, and outliers of the ultrasonic data. It is evident from the analysis that the meta-classifier demonstrates the highest consistency in accuracy and the least variability compared to other models.

**Figure 17:**
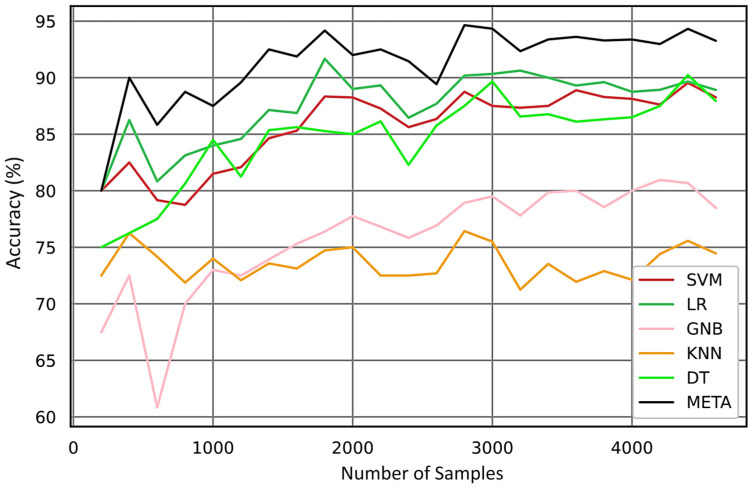
Accuracy curve vs. the number of samples (learning curve).

Accurately labeled data is crucial for the effectiveness of supervised learning algorithms in industrial fault diagnosis. Insufficient labeled data complicates model training and can lead to decreased accuracy. Additionally, the complexity of ultrasonic data, signal acquisition delays, and sample imbalances among faults present significant challenges in supervised diagnosis. Certain classifiers, like GNB, presuppose feature independence, which complicates the management of highly correlated data and hinders the attainment of optimal performance. Moreover, a limited dataset and uneven distribution of ultrasonic features may result in substantial predictive errors. Figure 17 highlights the issues arising from limited sample sizes, illustrating how insufficient data can diminish the predictive accuracy of classification models. This underscores the importance of larger and more balanced datasets for enhancing the efficacy of fault diagnosis models.

Figure 17 clearly demonstrates that the meta model can predict with high accuracy even with a limited number of samples, approximately 300. With an increase in the number of samples, the meta model consistently outperforms the other models in accuracy. 

**Figure 18:**
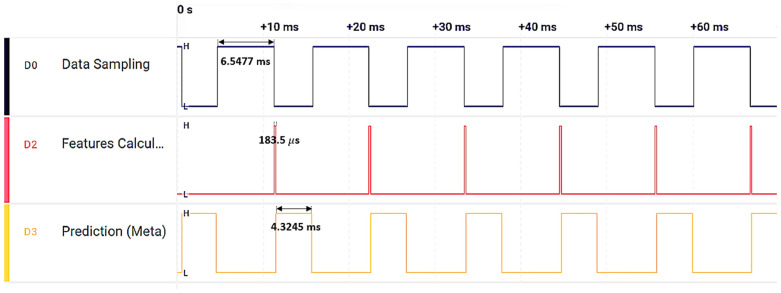
Execution timing analysis of the meta model implemented on the MCU.

In the final step, the micromlgen library in Python is used to extract the model and deploy it onto an nRF52840 64 MHz ARM Cortex-M4F MCU. The five optimal features, specifically kurtosis, zero crossing, skewness, shape factor, and peak index (as identified in Figure 11), are computed on the MCU to serve as inputs for five classifiers: GNB, LR, KNN, SVM, and DT. The outputs from these classifiers are then fed into the meta model, which executes in a total of 11 ms on this MCU—this includes 6.5 ms for data sampling, 183 µs for feature calculation, and 4.3 ms for the meta model prediction. These timings were verified using a logic analyzer, as depicted in Figure 18, confirming that our proposed model is highly suitable for real-time condition monitoring with relatively low computational requirements.

## 4. Conclusions

This work has introduced a precise ultrasonic industrial fault detection meta-classifier model designed for general-purpose applications, such as bearings and pipelines, utilizing contact sensors. This model is capable of determining and monitoring the health condition of industrial machinery. To assess the model’s performance, a k-fold CV strategy was employed to detect and classify ten different conditions within the ultrasonic signals. The reduction methods of RFE, PCA, t-SNE, and LDA were calculated, and RFE was chosen as the input for six classification models (namely SVM, GNB, LR, KNN, DT, and the meta classifier) to discern between ten distinct classes of irregular conditions. Each model’s performance was evaluated based on various metrics. The proposed meta classifier model demonstrated superiority with impressive results across multiple metrics, including ROC curves (i.e., achieving an AUC of 0.997), confusion matrices, k-fold validation (with 93% accuracy), and learning curves. Additionally, the model boasted a rapid execution time of just 11 ms on a 64 MHz Cortex-M4F MCU, confirming its suitability for real-time industrial fault monitoring applications. In future work, we aim to extend the model’s deployment capabilities to a wider range of MCUs to ensure broader applicability. We plan to incorporate IoT functionalities, enabling sophisticated remote data access and control mechanisms. Efforts will also be directed towards optimizing both power consumption and memory utilization, ensuring the model’s operation is both efficient and sustainable. Additionally, we will focus on the development of an optimized PCB design, specifically engineered to enhance the reliability and performance of the monitoring system within diverse industrial environments. These targeted improvements are intended to elevate the model’s practicality, adaptability, and efficiency in real-world applications.

## Figures and Tables

**Table 1:** Precision, recall, F1 measure, and accuracy of fault detection that are achieved by the ML models.

Classifier	Class	Precision	Recall	F1 Score	Accuracy	Classifier	Class	Precision	Recall	F1 Score	Accuracy
GNB	0	0.48	1.00	0.65	0.731	KNN	0	0.56	0.80	0.66	0.798
	1	0.57	0.45	0.50			1	0.62	0.58	0.60	
	2	0.89	0.83	0.86			2	0.86	0.84	0.85	
	3	0.97	0.93	0.95			3	0.92	0.88	0.90	
	4	0.50	0.46	0.48			4	0.68	0.71	0.69	
	5	0.82	0.92	0.87			5	0.87	0.87	0.87	
	6	1.00	0.99	1.00			6	0.98	1.00	0.99	
	7	0.88	0.95	0.91			7	0.84	0.91	0.87	
	8	0.66	0.58	0.62			8	0.82	0.70	0.75	
	9	0.91	0.22	0.35			9	0.80	0.57	0.66	
LR	0	0.58	0.95	0.72	0.817	DT	0	0.82	0.78	0.80	0.886
	1	0.76	0.63	0.69			1	0.72	0.74	0.73	
	2	0.91	0.82	0.86			2	0.93	0.91	0.92	
	3	0.92	0.90	0.91			3	0.96	0.99	0.97	
	4	0.80	0.57	0.66			4	0.79	0.85	0.82	
	5	0.85	0.93	0.89			5	0.88	0.92	0.90	
	6	0.99	1.00	0.99			6	1.00	0.99	0.99	
	7	0.76	0.95	0.84			7	0.99	0.96	0.97	
	8	0.66	0.53	0.59			8	0.89	0.85	0.87	
	9	0.84	0.66	0.74			9	0.84	0.82	0.83	
SVM	0	0.71	0.92	0.80	0.884	Meta	0	0.86	0.93	0.90	0.932
	1	0.84	0.67	0.75			1	0.95	0.83	0.88	
	2	0.96	0.84	0.90			2	0.96	0.88	0.92	
	3	0.98	0.97	0.97			3	0.98	1.00	0.99	
	4	0.83	0.84	0.83			4	0.89	0.96	0.92	
	5	0.88	0.95	0.91			5	0.90	0.96	0.93	
	6	0.99	1.00	0.99			6	1.00	1.00	1.00	
	7	0.97	0.97	0.97			7	1.00	0.97	0.98	
	8	0.84	0.88	0.86			8	0.88	0.91	0.90	
	9	0.89	0.79	0.84			9	0.92	0.89	0.91	

**Table 2:** Performance of SVM, GNB, LR, KNN, and meta-classifier models through 5-fold CV.

k-folds	SVM	GNB	LR	KNN	DT	Meta
Fold_1	0.877174	0.713043	0.811957	0.806522	0.878261	0.925140
Fold_2	0.893478	0.741304	0.856522	0.790217	0.890217	0.933696
Fold_3	0.902174	0.736957	0.858696	0.817391	0.871739	0.939135
Fold_4	0.879348	0.731522	0.835870	0.784783	0.876087	0.925581
Fold_5	0.885870	0.747826	0.833696	0.814130	0.916304	0.935870
Average	0.887609	0.734130	0.839348	0.802609	0.886522	0.931739

## Data Availability

Data are contained within the article.
